# Structural Evolution of Air-Exposed Layered Oxide Cathodes for Sodium-Ion Batteries: An Example of Ni-doped Na_*x*_MnO_2_

**DOI:** 10.1021/acs.chemmater.3c01196

**Published:** 2023-10-11

**Authors:** Gabriele Brugnetti, Claudia Triolo, Arianna Massaro, Irene Ostroman, Nicolò Pianta, Chiara Ferrara, Denis Sheptyakov, Ana Belén Muñoz-García, Michele Pavone, Saveria Santangelo, Riccardo Ruffo

**Affiliations:** † Dipartimento di Scienza dei Materiali, Università di Milano Bicocca, Milano 20125, Italy; ‡ Dipartimento di Ingegneria Civile, dell’Energia, dell’Ambiente e dei Materiali (DICEAM), Università “Mediterranea”, Via Zehender, Loc. Feo di Vito, 89122 Reggio Calabria, Italy; § Dipartimento di Scienze Chimiche, Università di Napoli Federico II, Napoli 80126, Italy; ∥ National Reference Center for Electrochemical Energy Storage (GISEL), Via G. Giusti 9, Firenze 50121, Italy; ⊥ Consorzio Interuniversitario per la Scienza e Tecnologia dei Materiali (INSTM), Via G. Giusti 9, Firenze 50121, Italy; # Laboratory for Neutron Scattering and Imaging, Paul Scherrer Institut, 5232 Villigen PSI, Switzerland; ∇ Dipartimento di Fisica “E. Pancini”, Università di Napoli Federico II, Napoli 80126, Italy

## Abstract

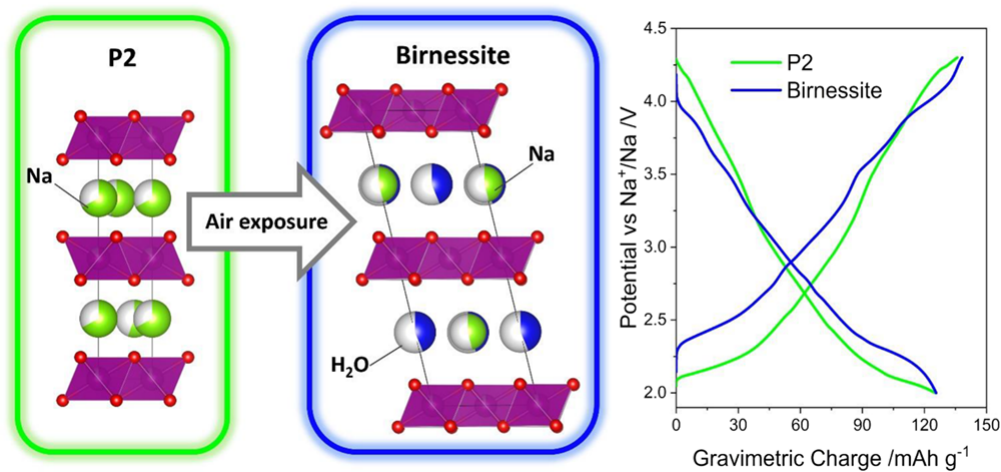

Sodium-ion batteries have recently aroused the interest of industries as possible replacements for lithium-ion batteries in some areas. With their high theoretical capacities and competitive prices, P2-type layered oxides (Na_*x*_TMO_2_) are among the obvious choices in terms of cathode materials. On the other hand, many of these materials are unstable in air due to their reactivity toward water and carbon dioxide. Here, Na_0.67_Mn_0.9_Ni_0.1_O_2_ (NMNO), one of such materials, has been synthesized by a classic sol–gel method and then exposed to air for several weeks as a way to allow a simple and reproducible transition toward a Na-rich birnessite phase. The transition between the anhydrous P2 to the hydrated birnessite structure has been followed via periodic XRD analyses, as well as neutron diffraction ones. Extensive electrochemical characterizations of both pristine NMNO and the air-exposed one vs sodium in organic medium showed comparable performances, with capacities fading from 140 to 60 mAh g^–1^ in around 100 cycles. Structural evolution of the air-exposed NMNO has been investigated both with ex situ synchrotron XRD and Raman. Finally, DFT analyses showed similar charge compensation mechanisms between P2 and birnessite phases, providing a reason for the similarities between the electrochemical properties of both materials.

## Introduction

The start of the ecological transition has given impetus to the production of electric vehicles (EVs). Lithium-ion batteries (LIBs) are considered the best option to power the next generation of electric vehicles. However, the limited availability of global lithium resources, coupled with their geographically uneven distribution, could drive up the price of lithium and slow the EV market penetration. Sodium-ion batteries (SIBs) appear to be a more sustainable alternative to LIBs in the field of energy storage, due to the greater abundance and wide geographical distribution of sodium in the Earth’s crust, its lower cost, and similar chemistry to lithium.^1^ Several companies, such as Contemporary Amperex Technology Co. Limited in China, Natron Energy based in Santa Clara, California, in collaboration with the Clarios Meadowbrook plant in Michigan, are already investing in the production of SIBs.

One of the key challenges of SIBs is to develop sustainable, low-cost, high-capacity, and stable cathodes. Recently, layered P2-type transition metal oxides Na_*x*_TMO_2_ (with TM = Ti, V, Cr, Mn, Fe, Co, Ni, or a combination of them) have gathered great interest because of their high theoretical capacity and energy density, attractive price, and environmental friendliness.^2−6^ They are constituted by sheets of TMO_6_ octahedra, providing 2D transport channels with a low barrier for Na^+^ ion diffusion between them.^2−6^ Especially Na_*x*_MnO_2_ has been the subject of extensive studies.^7−9^ However, despite expectations, the electrochemical performance for Na_*x*_MnO_2_ is often unsatisfactory. Upon Na^+^ intercalation, part of Mn^4+^ cations reduce to Mn^3+^ and MnO_6_ octahedra experience an anisotropic distortion with shortening/lengthening of two/four Mn–O bonds.^4^ The severe Jahn–Teller effect associated with the six-coordinated high-spin Mn^3+^ cations causes the transformation of the lattice structure from hexagonal (P2) to orthorhombic (P2′).^2,7,10^ The P2–P2′ transformation induces large lattice strain and Na^+^/vacancy order,^4^ which reflects in reduced sodium cation mobility and relevant capacity fading and further leads to structural collapse during repeated sodiation/desodiation cycles. The most commonly adopted strategy to mitigate cooperative Jahn–Teller distortion, suppress P2–P2′ phase transformation, and enhance structural stability of the oxide and mobility of the Na^+^ ion consists in the substitution of a proper amount (usually 0.05 < *y* < 0.2^2,7,10,11^) of Mn^3+^ Jahn–Teller centers with electrochemically inactive cations (such as Li^+^, Mg^2+^, Zn^2+^, Al^3+^) or active cations (such as Fe^3+^, Co^3+^, Ni^2+/3+^, and Cu^2+^),^2,6,10,12−16^ maintaining a single Na_*x*_Mn_1–*y*_M_*y*_O_2_ phase.^7^

A typical characteristic of various layered oxides is the reactivity toward molecules such as water and carbon dioxide. An important example is the work of Takada et al. on hydrated sodium cobalt oxide (e.g., Na_*x*_CoO_2_·yH_2_O), which was proven to be superconductive at temperatures below 4–5 K.^17−19^ For such a material (phase: P2 or P3), water (generally inserted in the structure through simple uptake from a moist atmosphere) is mandatory so to increase the interplanar distance and allow transition toward superconductive phases when temperature lowers.^20,21^

When talking about layered oxides as cathode materials, however, instability in air generally becomes an issue. This makes it necessary to store these materials in an inert atmosphere (Ar or N_2_) immediately at the end of the thermal stage of the synthesis or during the cooling process^22−24^ to avoid any possible contact with an atmosphere containing water or CO_2_.

Surprisingly, only a few studies deal with the effects of exposure to air and especially moisture, which always leads to a decrease in the specific capacities or in the stability upon cycling. The topic was first studied by Lu et al.,^25^ who discussed the stability of the Na_0.67_[Co_*x*_Ni_0.33–*x*_Mn_0.67_]O_2_ class of materials. No modifications were observed in the X-ray diffraction (XRD) pattern of Na_0.67_[Ni_0.33_Mn_0.67_]O_2_, pointing to its apparent stability in moisture/water. Conversely, changes were noticed for x values of 0.33 and 0.17. In particular, the shift of the (002) peak of the cathodic layered oxide to lower angles indicated an increase in the *c* parameter of the unit cell caused by water intercalation. For the sample synthesized with *x* = 0.33, the hydrated phase obtained was a pure layered one, and the hydration process was found to be reversible by heating the sample for several days at 200 °C. However, this study reported no consideration about the electrochemical performance of the hydrated materials.

A more recent study investigated the behavior of Na_0.67_[Ni_0.33_Mn_0.67_]O_2_ and Na_0.67_MnO_2_ cathodic materials exposed to air, once again by means of XRD analyses.^26^ In agreement with results previously reported by Lu et al.,^25^ no modification was observed in the XRD patterns of Na_0.67_[Ni_0.33_Mn_0.67_]O_2_, while the changes detected in the case of Na_0.67_MnO_2_ revealed an expansion in the *c*-axis of the cell and the formation of a pure hydrated phase. Such a phase was featured by the presence of water molecules in the MO_6_ octahedra interlayer, the dimensions of which increased from 5.5 to 7.1 Å. The pure hydrated phase exhibited the structure of Na-rich birnessite. It was also shown that the birnessite-structured phase was able to further intercalate water molecules, with consequent expansion of the *c* parameter of its cell, to form bauserite. The work by Zuo et al.^26^ also presented the results of the electrochemical characterization of the air-exposed cathodic materials. Surprisingly, despite the lack of evident changes in its XRD pattern, the air-exposed Na_0.67_[Ni_0.33_Mn_0.67_]O_2_ material exhibited reduced stability upon cycling. This was attributed to a partial extraction of Na-ions from the interlayer, caused by water and/or CO_2_. Similarly, the electrochemical performance of the hydrated Na_0.67_MnO_2_ phase was found to be worse than that of the sample not exposed to air, in terms of both Coulombic efficiency and stability.

Among different compositions of layered oxides, much attention has been devoted to Mn-rich phases, such as Na_0.67_Mn_0.9_Ni_0.1_O_2_ (**NMNO**) which are still more appealing due to the lower price and larger availability of Mn compared to Ni. This layered oxide, already an object of study in some previous studies,^11,27^ is interesting because of its high specific capacity, acceptable stability upon cycling and low content of nickel that, however, guarantees a good operating voltage. In the present work, the effect of exposing the P2-type Na_0.67_Mn_0.9_Ni_0.1_O_2_ (**NMNO**) cathodic material to air is investigated. In particular, a deep structural and morphological characterization of the pristine (P2 structure) and air-exposed (birnessite) phases has been carried out with the final aim of comparing the two phases electrochemically, as well as proposing a simple method to synthesize sodium-rich birnessite phases.

DFT calculations were performed to unveil the structural and electronic features occurring upon Na extraction from both phases. Structural evolution is addressed as the variation of Na/TM/H_2_O coordination upon desodiation, while magnetization and charge changes are evaluated to assess the charge compensation contributions during the cathode charge.

## Experimental Section

### Synthesis Pathway

Material with nominal composition Na_0.67_Mn_0.9_Ni_0.1_O_2_ was synthesized through the classic sol–gel method. Stoichiometric amounts of CH_3_COONa (purity: 99%, CAS No. 127-09-3), [CH_3_COO]_2_Ni (purity: 98%, CAS No. 6018-89-9), and [CH_3_COO]_2_Mn (purity: 98%, CAS No. 638-38-0) were dissolved in water. A 5% excess of sodium acetate was added to compensate for the loss of Na^+^ during the high-step process of the synthesis. Citric acid (purity: 99%, CAS No. 77-92-9) was then added to this solution, until an acid:metal-atom molar ratio of 3:1 was reached. A few droplets of ethylene glycol (EG, purity: 99%, CAS No. 127-21-1) were added to the so-obtained solution. After heating at 80 °C overnight, the obtained sol was dried at 300 °C for 2 h and then ground. The powder was pressed into a pellet (applied pressure: 12 atm) that was subsequently calcined in a platinum crucible in three steps, namely, 2 h at 350 °C, 2 h at 500 °C, and 9 h at 800 °C. The heating ramp between the different steps was 10 °C min^–1^. Half of the produced material was removed from the oven and placed in an argon-filled glovebox, while at a temperature of 400 °C, the remaining part, after cooling to room temperature (RT), was left in an open vial, thus exposed to air and moisture in the room atmosphere. The Na_0.67_Mn_0.9_Ni_0.1_O_2_ sample stored in an inert atmosphere, which maintained the pristine P2 structure, will be labeled as **NMNO_A**, whereas the sample exposed to air, finally showing a birnessite structure, will be labeled as **NMNO_B**.

### Physicochemical Characterization

#### X-ray Powder Diffraction

XRD patterns were acquired with a Rigaku Miniflex 600 diffractometer using copper K_α_ as the radiation source. Measurements were carried out in the angular range 5–70° with 0.02° step and scan rate of 1° min^–1^. The structural evolution of NMNO powders upon exposure to air was monitored by acquiring one diffractogram every week for 10 weeks. Apart from the as-synthesized powders, XRD patterns were also collected for a pristine electrode and two others at different states of charges, namely, desodiated at 20 and 60 mAh g^–1^ (the details relative to electrodes and cell preparations are reported in the following paragraphs). To carry out the analyses on the cycled electrodes, the active materials have been gently scratched from the current collector and subsequently inserted in a 0.3 mm quartz capillary. The obtained diffraction data were analyzed according to the Rietveld method using FullProf and Faults software.^28,29^

#### Neutron Diffraction

NMNO_A and NMNO_B powders were further characterized through neutron powder diffraction to get insight into the sodium and water content. Neutron powder diffraction data were collected at the HRPT beamline at the Swiss Spallation Neutron Source SINQ in PSI.^30^ Powders were loaded into a vanadium sample holder to minimize the background. Measurements were carried out at RT using 1.49400 Å wavelength in the 4–165° angular range with step size 0.05°.

#### Synchrotron X-ray Powder Diffraction

To get insight into the structural changes due to the sodiation and desodiation process, selected NMNO_B samples from dismantled cells were analyzed through synchrotron powder diffraction. Data were collected at RT using wavelength λ = 0.20735 Å at the P02.1 beamline at the PETRAIII – DESY facility. All samples were measured on a beamline area detector: Varex XRD 4343CT (150 × 150 μm^2^ pixel size, 2880 × 2880-pixel area, CsI scintillator directly deposited on amorphous Si photodiodes). The detector calibration was performed by using LaB_6_ (NIST 660c) as a standard; the obtained data were exploited for the creation of the instrumental resolution file. The obtained 2D data were then processed and integrated to obtain 1D traditional diffraction patterns.

#### Micro Raman Analysis

Micro Raman analysis was carried out on the NMNO_B active material and on as-prepared and cycled electrodes, using an NTEGRA—Spectra SPM NT-MDT confocal microscope coupled to a solid-state laser operating at 532 nm, the intensity of which can be varied by means of a variable-step neutral filter. Measurements were performed in air at RT in reflection mode: the incident and scattered light from the sample was passed through a 100× Mitutoyo objective (NA = 0.75). Finally, the scattered signal was dispersed by 600 lines mm^–1^ grating and detected by a cooled ANDOR iDus CCD Camera.

#### Thermogravimetric Analysis

Thermogravimetric analyses (TGA) were performed with a Mettler Toledo TGA/DSC-1 instrument under an Ar atmosphere, with a heating rate of 5 °C min^–1^ in the temperature range 30–1000 °C.

#### Elemental Analyses

The stochiometric coefficients of sodium, manganese, and nickel were evaluated through ICP-OES analyses using an Avio 220 Max Spectrometer. Samples were dissolved in aqua regia at 100 °C and then diluted so as to fit the detection limits. Powders were also washed in ethanol and then dried out at 80 °C so as to get rid of all soluble compounds that might have been formed during air exposition. Carbon and hydrogen were analyzed using an Elemental-vario MACRO cube analyzer.

#### Scanning Electron Microscopy

Morphological analysis was performed with an SEM Zeiss Gemini electron microscope. To avoid the charging effects during measurements, the samples were preliminarily metalized with chromium. Before analysis, the tested samples were washed several times with the electrolyte’s solvent.

### Electrochemical Tests

#### Electrodes Preparation

Electrode formulations were prepared by mixing active material, conductive carbon (Super-P), and binder (PVDF) in an 8:1:1 mass ratio in n-methyl-2-pyrrolidone; an Ika UltraTurrax T10 disperser was used for this purpose. The so-obtained slurries were casted on an aluminum foil with a thickness of 20 μm, dried at 80 °C under vacuum, and then pressed with a hydraulic press applying a 2 tons pressure. The obtained active mass load was 1.5–2 mg cm^–2^.

#### Chemical and Electrochemical Tests in the Organic Electrolyte

Electrochemical tests were carried out by using a BioLogic VSP-300 potentiostat/galvanostat. Hohsen CR2032 coin cells were assembled by testing the active material formulation as the working electrode, metallic Na as the counter electrode, glass microfiber (Whatman) as the separator and 1 M NaClO_4_ in propylene carbonate (PC) with 2% fluoroethylene carbonate (FEC) as the electrolyte. Measurements were carried out testing the half-cells with a gravimetric current of 10 mA g^–1^ in the potential interval 2.0–4.3 V vs Na^+^/Na.

Water content in the electrolyte before and after contact with NMNO_B has been measured through Karl Fischer titration. Specifically, an electrode (area = 2 cm^2^) has been soaked in 100 μL of the electrolyte for 24 h. The solution was then taken and titrated using a Metrohm 899 Coulometer, and the obtained value was compared with that measured in the pristine electrolyte.

#### Diffusion Coefficient Calculation

The diffusion coefficient was determined by the galvanostatic iterative titration technique (GITT) by charging or discharging the material for 15 min at 10 mA g^–1^. At the end of every step, electrochemical impedance spectroscopy (EIS) was performed after 30 min of rest. A sinus amplitude of 10 mV and frequencies varying from 1 MHz to 100 mHz were used. The diffusion coefficient was estimated through formula 1, where τ is the pulse duration, *n*_m_ is the number of moles of active material, *V*_m_ is molar volume of the electrode as calculated from the tap density, *S* is the area of the electrode, Δ*E*_s_ indicates the change in the steady-state voltage resulting from the current pulse, and Δ*E*_t_ stands for the total potential variation during the constant-current step, eliminating the IR drop, as evaluated by fitting EIS data.
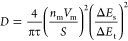
1

#### Tap Density Measurement

To evaluate tap density, at least 500 mg of active material was put in a 1 mL syringe after having removed the syringe piston. The syringe was then tapped on the laboratory bench several times and the volume was measured according to the procedure reported in the literature for similar systems.^31−33^

### Computational Details

Spin-polarized density functional theory (DFT) calculations are performed with the DFT+U Hubbard-like correction scheme to overcome the large self-interaction error (SIE) that affects DFT when applied to mid-to-late first-row TM oxides with tightly localized d-electrons.^34−36^ Projector-augmented wave (PAW) potentials and plane wave (PW) basis sets have been used, as implemented in the Vienna Ab-initio Simulation Package (VASP) code (version 5.4.4).^37^ For all of the calculations, the following PAW potentials have been considered: Na_pv [Be]2p^6^3s^1^; Mn [Ar]3d^7^; Ni [Ar]3d^10^; O [He]2s^2^2p^4^; H 1s^1^.^38^ We have employed the Perdew–Burke–Ernzerhof (PBE) exchange-correlation functional with *U*_eff_ = 4.0 eV parameter for both Ni and Mn atoms and added the D3-BJ dispersion correction to account for van der Waals (vdW) interactions that play a crucial role in layered structures.^39−43^ A kinetic energy of 750 eV and Γ-centered 1 × 1 × 1 k-points sampling mesh have been used; these values ensure converged energies within 3 meV/f.u. with respect to the PW basis set size and Brillouin zone sampling, respectively. For all the calculations, the convergence threshold for energy has been set to 10^–5^ eV. The structural models for the P2 (NMNO_A) and birnessite (NMNO_B) phases consist of, respectively, a 220 atoms-containing 5 × 3 × 1 supercell of Na_0.67_Mn_0.9_Ni_0.1_O_2_ within the *P*6_3_/*mmc* space group and a 300 atoms-containing 3 × 5 × 1 supercell of Na_0.5_Mn_0.9_Ni_0.1_O_2_ · 0.5 H_2_O within the *C*2/*c* space group (see Figure S1a). For both phases, the mixed occupancy of Ni/Mn at the corresponding atomic sites results in TM disorder,^44,45^ which can be simulated via the special quasi-random structure (SQS) approach as implemented in the Alloy Theoretic Automated Toolkit (ATAT) code.^46−49^ Na atoms have been placed in edge and face sites with Na(e)/Na(f) ratio being equal to 2 for each sodiation state.^50^ Lattice constants and atomic positions *x* = 0.67, 0.57, and 0.47 for P2 and *x* = 0.50, 0.40, and 0.30 for the birnessite phase have been fully relaxed until the maximum forces acting on each atom were below 0.03 eV/Å (see Figure S1). The net magnetic moment on each atom is obtained as the difference in the up and down spin channels integrated within a sphere with a Wigner-Seitz radius for each atom type (default values from VASP are used).

## Results and Discussion

### XRD Analysis Results

**Figure 1:**
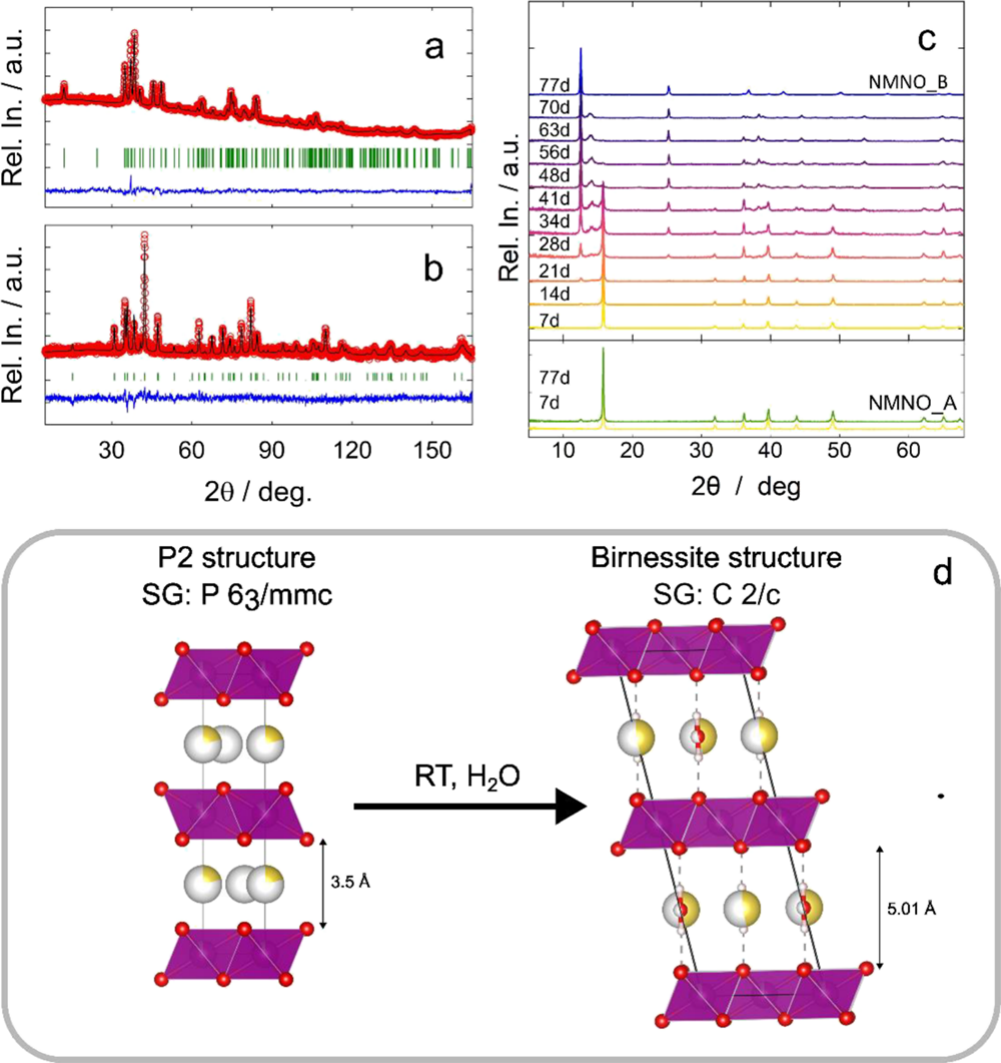
(a) Neutron diffraction patterns and results of Rietveld refinements for samples NMNO_A and (b) NMNO_B after complete transition to the birnessite phase; (c) time-evolution of the XRD patterns from NMNO_A (bottom panel) to NMNO_B (top panel); (d) crystal structure of P2-type layered oxide and birnessite.

The results of structural analyses by neutron and X-ray diffraction are displayed in Figure 1a−c, respectively. XRD analysis proves that the target material (NMNO_A) was successfully synthesized as a P2 phase, with the peculiar, layered structure belonging to the space group *P*6_3_/*mmc* (Figure 1d). No impurity-related minor peaks were detected in the XRD pattern (bottom panel of Figure 1c). Moreover, by comparing the diffractograms recorded after the first and 11th week (bottom panel of Figure 1c), no obvious differences can be observed, which proves that the material stored in an inert atmosphere maintained its initial structure.

On the contrary, the patterns of the sample exposed to air (NMNO_B) undergo a progressive evolution by lowering the intensity of the P2 phase-related peaks and the appearance of new peaks. Particularly indicative is the main P2-peak at 15°, which progressively decreases in intensity until, after 70 days, it disappears completely. This is compensated by the growth of new peaks, the more intense at 12°, which gradually increase, highlighting the occurrence of a phase transformation driven by the intercalation of water molecules between the sheets of MO_6_ octahedra. Once the transition is completed, the pattern of NMNO_B can be associated with the sodium-rich birnessite structure (JCPDF Card No. 01-073-9669) (Figure 1d). This phase is the same one identified by Zuo et al.,^51^ for a similar composition without nickel doping. The two layered structures (P2 and birnessite) essentially differ from each other only for the MO_6_ interlayer spacing, the Na-ions positioning within the layers, and the presence of interlayer water molecules (Figure 1d), while the (Mn/Ni)–O connectivity and (Mn/Ni)O_6_ unit relative orientation are the same. The P2-to-birnessite evolution probably involves the formation of at least one intermediate phase that has not been isolated (i.e., in no one of the registered patterns can be found as a single phase), as evident from the peak at 14° in the 34–70 d related patterns in Figure 1a. The material with birnessite structure was found to be stable under ambient conditions. This is not surprising as birnessite is a known mineral, also found in nature and several compositions have been reported as well as hexagonal, monoclinic, and triclinic structures.^52,53^ Remarkably, all acquired diffraction patterns are characterized by sharp symmetric reflections, indicating the highly crystalline nature of both pristine P2 and birnessite-transformed materials, while for the 28–41 days-related patterns, some broader peaks are observed, suggesting that some of the intermediate states are not ordered. It is worthwhile noticing that obtaining highly crystalline P2 material is not a difficult task, while many of the previous reports on synthetic birnessite present blurred diffraction patterns, as typical of disordered layered materials.^54−56^ The simple method presented here (aging under ambient conditions) allows us to obtain a final phase without significant changes in the Na, Mn, and Ni composition (as discussed below) and with a crystallinity degree comparable to that of the starting material. Thus, it represents the optimal choice for a direct comparison of the two materials from the point of view of the electrochemical performance.

As already mentioned, the transition from P2 to the birnessite structure was already investigated for a similar composition with a lower content of sodium and a Ni content of 0 and 33%.^51^ A further evolution of the structure was reported, ultimately leading to the formation of bauserite.^51^ On the contrary, in the present case, no further expansion of the structure to form bauserite phase^51^ was observed by immersing part of the powders in water for 1 week, which demonstrates the stability of the structure obtained upon prolonged exposure to air. Therefore, sample NMNO_B was chosen for all subsequent physicochemical and electrochemical characterization.

### Neutron Diffraction Analysis Results

In order to infer additional information, neutron diffraction data were collected for samples NMNO_A and NMNO_B (after complete conversion to birnessite), and Rietveld refinements were carried out. The results obtained are reported in Figure 1a,b and Table S1. The refinement for NMNO_A leads to cell parameters *a* = 2.87161(25) Å and *c* = 11.18154(106) Å, in good agreement with the previous report for similar compositions.^11,27^ The Mn:Ni occupancies obtained (0.87:0.13 ratio) confirm the effectiveness of doping with the considered synthetic procedure. The refined Na content (0.4) is lower than that for nominal composition, in line with other values reported in the literature.^51^

The Rietveld refinement for NMNO_B, based on the use of the *C*2/*c* space group, leads to cell parameters *a* = 5.01208(84) Å, *b* = 2.89406(41) Å, *c* = 14.45890(187) Å, and β = 103.23717(1025)°. A good agreement with previous reports considering the *C*2/*m* space group with cell parameters *a* = 5.015 Å, *b* = 2.901 Å, *c* = 7.246 Å, and β = 103.10° is obtained. Nevertheless, the removal of the basal reflection plane, implying the change in the space group, has been introduced to account for the presence of water molecules within the Na layers. Indeed, the water molecules are located in the same plane as the sodium ion (Figure 1d), with the site for oxygen species identified in (0, 0.3070, 0.25). This model has been necessarily introduced to statistically account for the copresence of water molecules with random orientation and sodium ions and it is the best option based on the available neutron data that do not allow for a deeper analysis. The refined Mn:Ni ratio leads to the value 0.89:0.11, very similar to those obtained for NMNO_A. This result is not surprising as the composition in terms of Mn and Ni is not expected to change with the structural phase transition. The Na content is estimated to be 0.46, again in nice agreement with the Na content of NMNO_A, suggesting that the phase transition does not involve ion exchange. The water content obtained from the refinement is 0.44 units per formula, corresponding to the composition Na_0.46_(H_2_O)_0.44_Mn_0.89_Ni_0.11_O_2_. The inspection of the neutron pattern collected for NMNO_B (Figure 1c) and the comparison with the one obtained for NMNO_A (Figure 1b) reveals a strong increase and a typical modulation of the background; this can be attributed to the presence of significant amounts of natural hydrogen in the material, giving origin to strong incoherent scattering, that can be associated with the presence of extra protons/water in the samples.

### Thermal and Elemental Analyses Results

**Figure 2:**
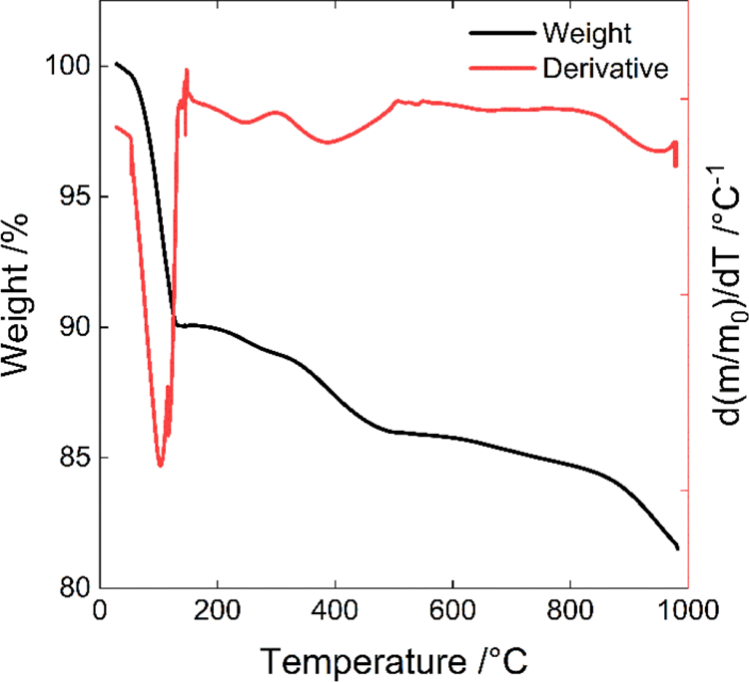
Thermal profile and its derivative were obtained for sample NMNO_B.

This quantification was confirmed by the investigation of NMNO_B by TGA in air (Figure 2).

Three main weight losses can be identified in the thermal profile. The first weight loss (10 wt %), occurring at temperatures lower than 120 °C, is related to the desorption of water from the material, which demonstrates that the material is highly hygroscopic. In the derivative curve, this weight loss corresponds to the big, sharp peak centered at 102 °C. The second reduction of weight (4 wt %), attributable to the loss of water intercalated among the MO_6_ layers, occurs between 120 and 450 °C. It gives rise to two broad peaks centered at 247 and 388 °C in the weight derivative curve. The temperature range in which this loss takes place is in agreement with the data presented in a previous study on similar materials.^25,51^ Another possibility is that part of this loss can be attributed to the presence of unburned organic compounds from the synthesis. This is possible because of the palletization of the reactants. Finally, the third loss of weight occurs at temperatures higher than 900 °C (peak at 950 °C in the weight derivative curve), and it is probably attributable to the material decomposition.

To have a more precise understanding of the chemical composition (Table S3) of both NMNO_A and NMNO_B, elemental analyses (ICP, CHNS), and a TGA of NMNO_A, were performed. For both samples, ICP analyses revealed the presence of 0.9 and 0.1 equiv of manganese and nickel, respectively, and a sodium content varying from 0.65 to 0.52 upon air exposition. The decrease in the sodium content is probably compensated by partial oxidation of manganese and/or nickel. The discrepancies between the values obtained by ICP and those refined from diffraction analyses are probably due to the presence of small amounts of impurities (e.g., sodium hydroxides or carbonates), which were not enough to be included in the Rietveld refinement algorithm.

CHNS analyses showed the presence of both carbon and hydrogen, the second of which increases from 0.144 to 1.38 wt % upon air exposure. While the increase in the water content can be attributed to water uptake, it should be noted that the constant value of the carbon content likely means that no carbon dioxide is adsorbed by such material.

Finally, from the NMNO_A TGA shown in Figure S3, it is possible to observe the absence of adsorbed water (i.e., no weight loss before 120 °C). Also, the two broad losses between 120 and 450 °C are substituted by an almost monotonic loss that starts around 300 °C, for a total of 5.2 wt %. This is probably related to the unburned organic compounds from the synthesis, which remain almost unchanged in NMNO_B.

The composition of NMNO_B, calculated based on these data, is Na_0.52_(H_2_O)_0.3_Mn_0.9_Ni_0.1_O_2_, where 0.3 is the molar fraction of water intercalated among the layers. In addition, around 0.6 molecules of water per unit formula are adsorbed on the surface of the cathodic material. Thus, these results confirm the indications that emerged from the neutron diffraction analysis. For this reason, before the electrochemical tests were performed, the NMNO_A sample was dried at 100 °C under vacuum overnight.

### Morphological Analyses Results

**Figure 3:**
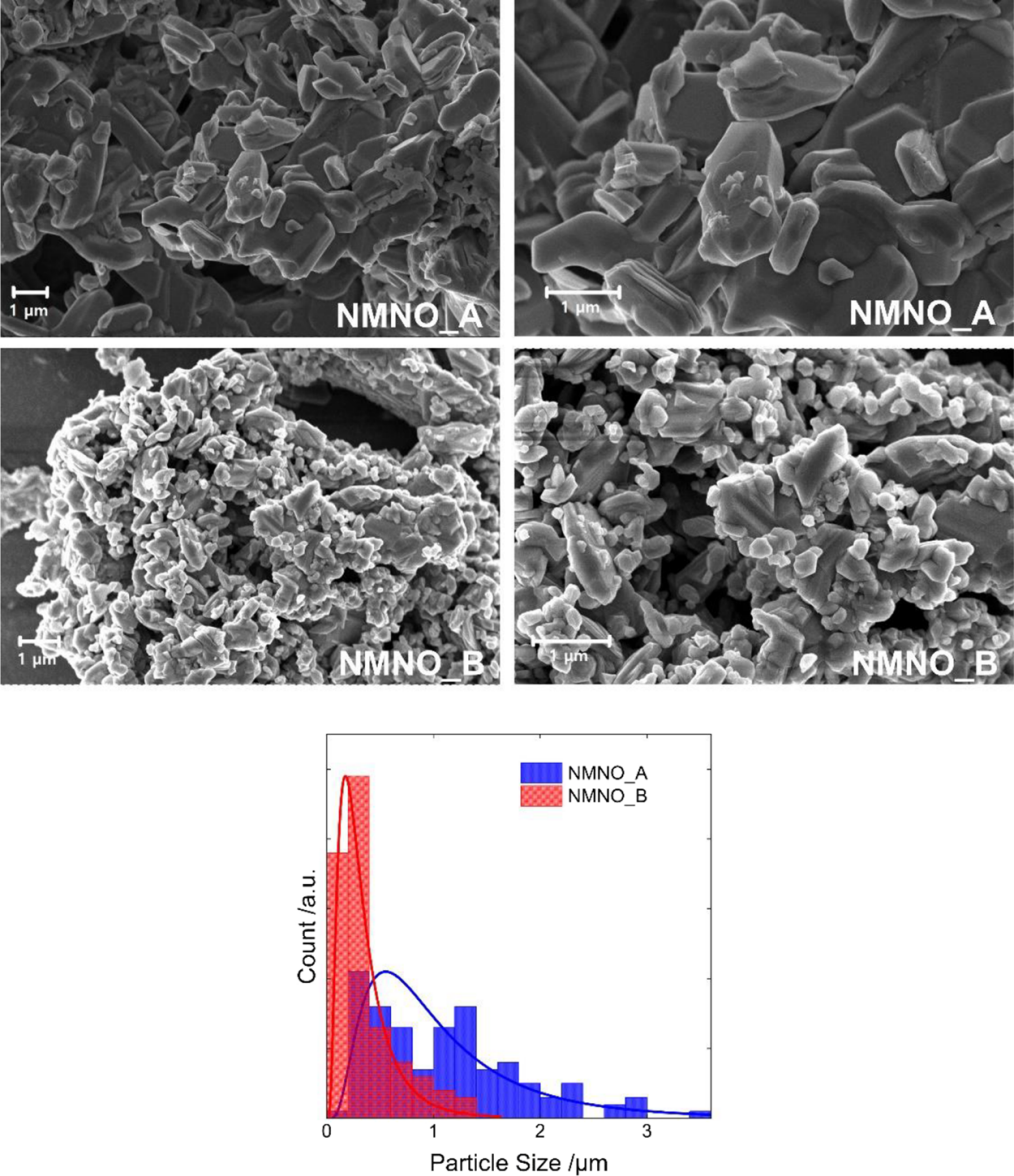
SEM images of the two different samples and analysis of the particles’ size distribution.

The morphologies of NMNO_A and NMNO_B were evaluated by means of SEM. From Figure 3, it is possible to observe that the samples have similar morphologies. As typical of this class of materials,^11^ they are constituted by tablet-like particles, characterized by very smooth surfaces. In some cases, the active material particles present a hexagonal shape with well-defined edges, as expected from the structural data. The main difference between the two samples lies in the particle size. In NMNO_B, particles are generally smaller than those in NMNO_A, as expected because of water intercalation which induces particle cracking in layered structures.^51^ Going into more detail, the NMNO_A particles have an average edge length of 1.1 μm, whereas at the end of the water intercalation process, this value is reduced to 0.36 μm. In the latter sample, only a few particles with dimensions>1 μm can be detected, whereas particles with dimensions >3 μm are present in NMNO_A. Moreover, a slight local delamination of the sample exposed to air along the *c*-axis is observable, in agreement with the literature.

### Electrochemical and Spectroscopic Characterization

#### Electrochemical Performances in Organic Media

**Figure 4:**
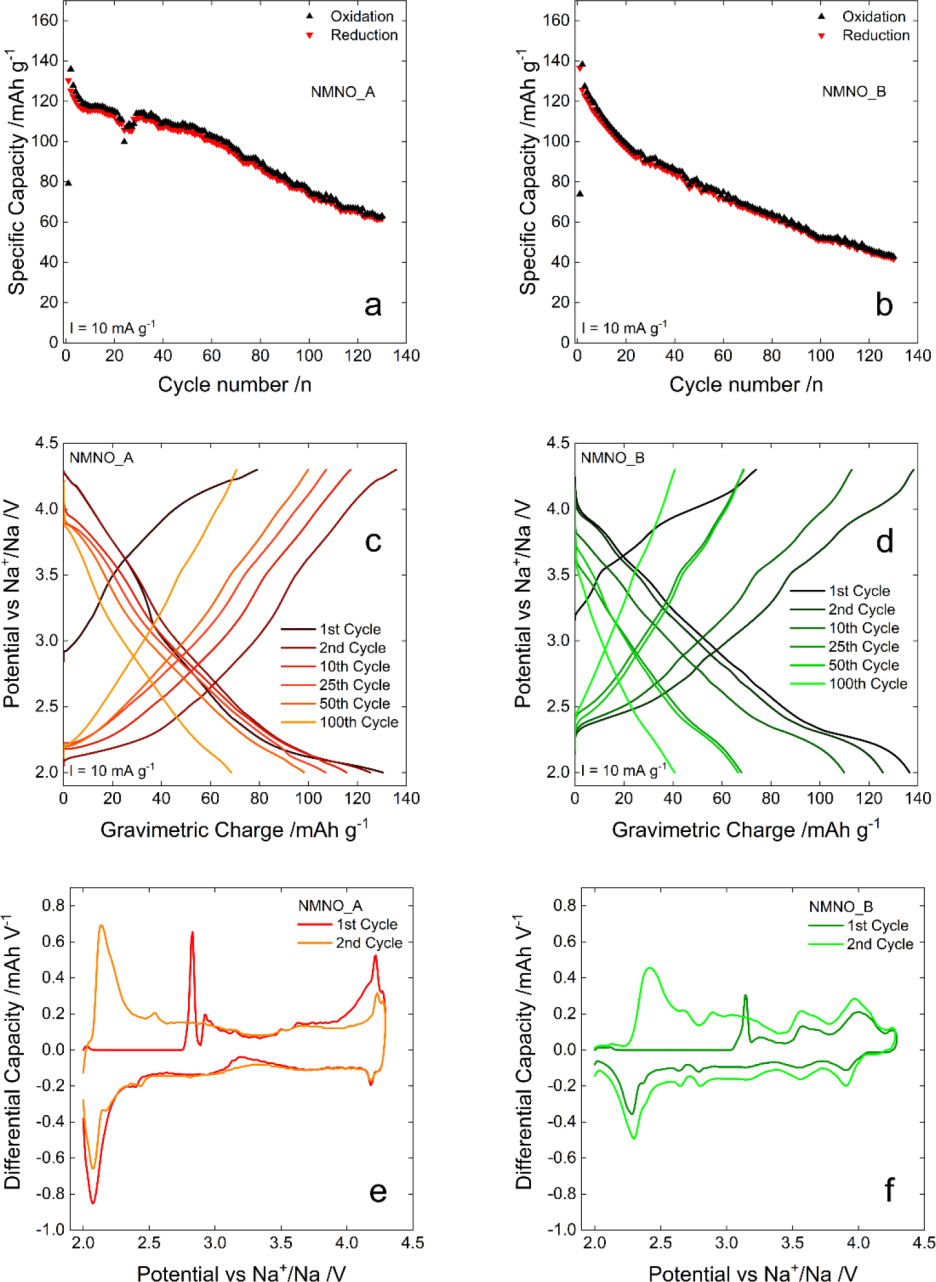
Results of the electrochemical test on electrodes based on NMNO_A and NMNO_B. (a, b) Cyclic performance and (c, d) galvanostatic reduction/oxidation cycles at 10 mA g^–1^; (e, f) differential capacity plots.

Figure 4 compares the results of the tests carried out on the two investigated active materials in the organic electrolyte, where cycling stability (Figure 4a,b), rate capability (Figure 4c,d), and differential capacity plots obtained from the first two cycles (Figure 4e,f) are shown to evaluate the desodiation/sodiation mechanism.

Electrodes based on NMNO_A and NMNO_B show comparable specific capacities in the first sodiation cycle (130 and 136 mAh g^–1^ for NMNO_A and NMNO_B, respectively) with values in perfect agreement with the literature on the P2 structure.^27^ Conversely, they exhibit different capacity retention and desodiation/sodiation mechanism, as reasonably expected, based on the different interlayer distance in their lattice and composition (i.e., the presence of water between the MO_6_ layers). In the galvanostatic reduction/oxidation cycles at a current density of 10 mA g^–1^, the cathodic specific capacity of NMNO_A decreases from 130 mAh g^–1^ in the first cycle to 118 mAh g^–1^ in the fifth cycle, and then it maintains a value higher than 100 mAh g^–1^ for 60 cycles, thus showing a capacity retention of 77%. Subsequently, the electrochemical performance of NMNO_A worsens, and at the end of the 130th cycle, it delivers only 72 mAh g^–1^ that corresponds to a capacity retention of 55%. Then, the specific capacity undergoes a continuous decrease without any stabilization. After 60 cycles, the retention is only 52%. After 100 cycles, the electrode delivers a specific capacity lower than 60 mAh g^–1^ and, at the end of the 130th cycle, the specific capacity of the material exposed to air drops to 49 mAh g^–1^.

The desodiation/sodiation profiles of the two electrodes (Figure 4c,d) exhibit some differences, even if both present a pseudocapacitive curve, with a plateau between 2.0 and 2.5 V. In NMNO_B, the position of this plateau is shifted at higher potential both in desodiation and sodiation. The shift could originate from the interaction between the Na^+^ ions and the O anions of water that increases the energy required to remove the mobile ions. This different position is better visible in the differential capacity plots relative to the first two cycles (Figure 4e,f). Although both materials present a very broad pattern in the 2.0–2.5 V potential region, reflecting a pseudocapacitive behavior, the oxidation and reduction peaks of NMNO_A are located at lower potentials than those of NMNO_B. Furthermore, NMNO_A presents an oxidation peak and a reduction peak at potential values near 4.25 V, a region where NMNO_B does not show reactivity.

For NMNO_A, the mean operating potentials, as calculated over the first 20 cycles, are 3.06 and 2.82 V for the oxidation and reduction process. The values obtained for NMNO_B (3.22 and 2.73 V, respectively) indicate that water insertion between the MO_6_ layers causes an increase in the oxidation potential and a lowering in the reduction one. Such a difference also affects the energetic efficiency, the average value of which, calculated from the same data, is 91.2% for NMNO_A and 83.0% for NMNO_B. The larger difference between mean oxidation and reduction operating potentials for NMNO_B accounts for its diminished energetic efficiency.

Karl Fischer titration on the electrolyte before and after being in contact with an electrode of NMNO_B for 24 h was, in both cases, 25 ppm. This means that no water is released from the electrode, proving its stability. As discussed later in the text, DFT results will show how intercalated water in the birnessite phase can establish favorable interactions with the TMO_2_ layer, thus revealing the origin of structural stability for the birnessite phase.

A SEM image of the surface of NMNO_A and NMNO_B electrodes after 75 cycles is reported in Figure S4 in the Supporting Information. All electrode components are visible in the SEM images, namely, conductive carbon and binder, both with nearly spherical shape and size around 50 nm, and active material particles featured by regular edges, as in the pristine material, which proves their unaltered crystallinity. No evidence of delamination is detected in the samples.

#### Ex Situ Diffraction and Spectroscopic Analyses

**Figure 5:**
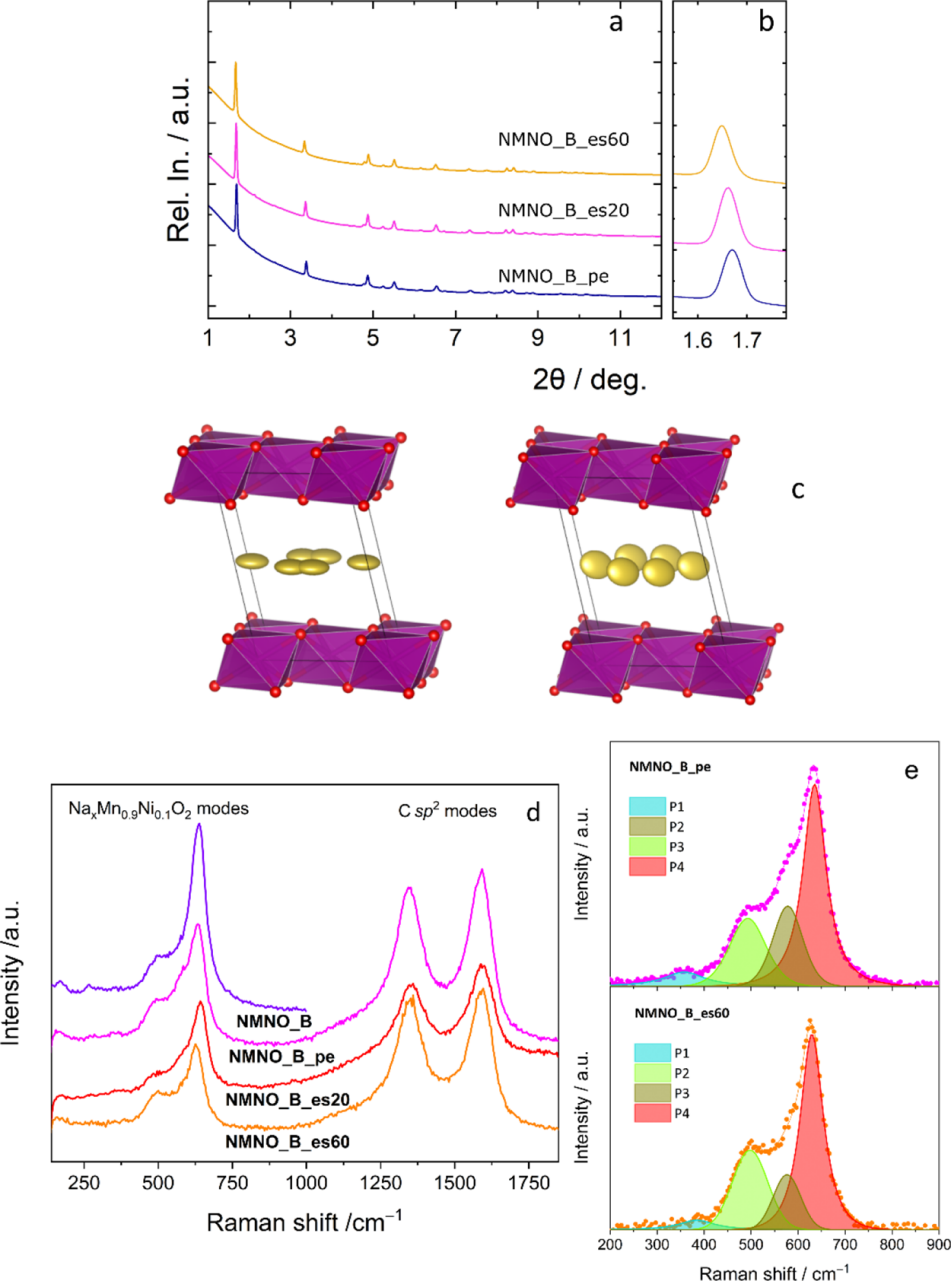
(a, b) Ex situ synchrotron X-ray diffraction data for the NMNO_B pristine electrode, the NMNO_B_es20, and NMNO_B_es60 cycled samples (a) with focus on the main reflection (b); (c) anisotropic displacement factors for Na in the refined NMNO_B pristine electrode and NMNO_B_es60 structures; (d) ex situ micro-Raman spectra for pristine and cycled electrodes (the spectrum of active material is also reported for comparison); (e) fits to the spectra

Differently from the P2 structure, the behavior of which upon sodiation and desodiation has already been extensively studied,^11,27^ the birnessite system has not received the same attention. For this reason, the electrode structure was investigated through ex situ synchrotron X-ray diffraction, and the results are reported in Figure 5. Figure 5a, Table S2, and Figure S2 display the diffraction patterns collected on the pristine electrode (NMNO_B_pe) and two electrodes at different cell cycling stages during desodiation (NMNO_B_es20 and NMNO_B_es60). The lack of differences in the profiles indicates that no phase transition occurs. Only shifts in the peak positions are observed. They are related to the lattice relaxation upon sodium extraction. The Na content of hydrated Na_*x*_Mn_0.9_Ni_0.1_O_2_ progressively decreases from *x* = 0.52 in NMNO_B_pe, to *x* = 0.47 in NMNO_B_es20, and to *x* = 0.44 in NMNO_B_es60. The *x* value in NMNO_B_pe is slightly larger than that inferred from neutron diffraction (0.4), but smaller than the nominal one (0.67). Based on the charge, *x* should be 0.58 for NMNO_B_es20 and 0.43 for NMNO_B_es60. Although all the values obtained from the refinements are different from the nominal ones, the evolution of the composition follows the expected trend. The variation of the cell parameters indicates that the desodiation induces a distortion of the cell; *a* and *b* shrink, while *c* expands; correspondingly, the overall volume is only slightly modified. This finding is consistent with the presence of water molecules that do not allow for a large reduction in the distance between the layers even for significant changes in the sodium ion content.

Further analyses were carried out to deeply investigate the structural evolution during cycling. The analysis of the isotropic displacement factors refined separately for Na, (Mn/Ni), and O reveals an anomalous variation for Na ions. The isotropic displacement factors for (Mn/Ni) and O (∼0.97 and ∼1.21 Å^2^, respectively) show no evolution with changing Na content, whereas the isotropic displacement factors of sodium increase from 4.9 Å^2^ in the pristine electrode to 5.7 and 9.4 Å^2^ in NMNO_B_es20 and NMNO_B_es60, respectively. This suggests that the degree of disorder in the Na layer is extremely high and increases upon desodiation, as expected. For this reason, after refining the Na occupancies, these were kept fixed, and anisotropic displacement factors were considered only for Na-ion; the results obtained are reported in Figure 5c. As expected, the refined ellipsoids suggest a high tendency of sodium to displace within the *ab* plane, and this tendency is greater for the less sodiated sample where a larger number of empty sites is available for Na ions.

Finally, a progressive asymmetric broadening of the main (001) reflection of the birnessite structure at 1.68° was found to accompany the desodiation process (Figure 5b). This phenomenon has been already reported for layered systems and particularly for intercalation-type electrode materials.^57−63^ The diffraction feature is associated with the presence of 2D defects and stacking faults for the sequence of (Mn/Ni)O_2_ and Na/H_2_O layers. For this reason, the data relative to NMNO_B_es60 were analyzed through the Faults software allowing to account for this kind of defects and improve the overall fit quality. The results indicate that the main source of disorder is the partial sliding of the (Na/H_2_O) layer with respect to the (Mn/Ni)O_2_, which leads to the distortion of the Na coordination with oxygen ions.

Thus, to summarize, as the nominal sodium content of hydrated Na_*x*_Mn_0.9_Ni_0.1_O_2_ decreases from *x* = 0.52 to 0.43 (corresponding to 0.52 and 0.44 refined *x*-values, respectively), the birnessite structure does not undergo a phase transition, but it relaxes through a distortion involving the variation of structural parameters. With decreasing occupancies of sodium sites, Na ions are characterized by greater displacement factors, probably related to their higher mobility and to the larger availability of free sites. The formation of 2D defects accompanies this change; it involves the sliding of the (Na/H_2_O) planes with respect to the (Mn/Ni)O_2_ layers, which allows minimizing of the interlayer repulsion.

Figure 5d displays the results of Raman scattering measurements on pristine and cycled electrodes. Due to the heterogeneous nature of the electrodes, several spectra were collected from random locations in each specimen and averaged to have a reliable picture of it. The Raman fingerprint of amorphous carbon is visible in the higher frequency region of the spectra (>1000 cm^–1^), namely the D- and G-bands at ∼1346 and ∼1590 cm^–1^, respectively. The phonon modes arising from hydrated Na_*x*_Mn_0.9_Ni_0.1_O_2_ are detected in the lower frequency region. The spectrum of the active material (NMNO_B) is dominated by the very intense band at 635 cm^–1^. The band originates from the *A*_1g_ symmetric (Mn/Ni)–O stretching vibration of (Mn/Ni)O_6_ octahedra along the *c*-axis (υ_1_).^64−67^ Other bands associated with the (Mn/Ni)–O vibrations and having lower intensity are detected at lower frequencies. They arise from the (Mn/Ni)–O stretching vibration in the basal plane of [(Mn/Ni)O_6_] sheets at 580 cm^–1^ (υ_2_), and from the *E*_g_ (Mn/Ni)–O vibration of birnessite structure at 493 cm^–1^ (υ_3_).^64,66^ The very weak contributions at ∼360 cm^–1^ (υ_4_) and ∼280 cm^–1^ (υ_5_) are ascribed to the asymmetric stretching vibration of the Na^+^ ions.^64,66^

The lower frequency region of the spectra of the pristine and cycled electrodes does not significantly differ from the spectrum of the active material, confirming the preservation of the birnessite structure. However, in order to infer more detailed information on the structural changes promoted by the desodiation process, the region where the most intense contributions originating from hydrated Na_*x*_Mn_0.9_Ni_0.1_O_2_ are detected was fitted to four bands (Figure 5e). The downshift of the *A*_1g_ band (from 635 cm^–1^ in NMNO_B_pe to 629 cm^–1^ in NMNO_B_es60) accompanies the decrease in the sodium content. As the vibration occurs along the *c*-axis, this change is indicative of the tensile strain introduced by the expansion of the *c* parameter. Moreover, the intensity of the *A*_1g_ band increases relative to that of the band associated with the (Mn/Ni)–O stretching in the basal plane of [MnO_6_] sheets. This change points to the existence of smaller constraints in the (Mn/Ni)–O stretching vibrations along the *c*-axis and is consistent with the decreased sodium content in the interlayer region. Also, the weakening of the contribution due to the vibration of the Na^+^ ions at ∼360 cm^–1^ is consistent with the decrease of *x*.

#### Electrochemical Performances in Organic Media with Smaller Cut Off

**Figure 6:**
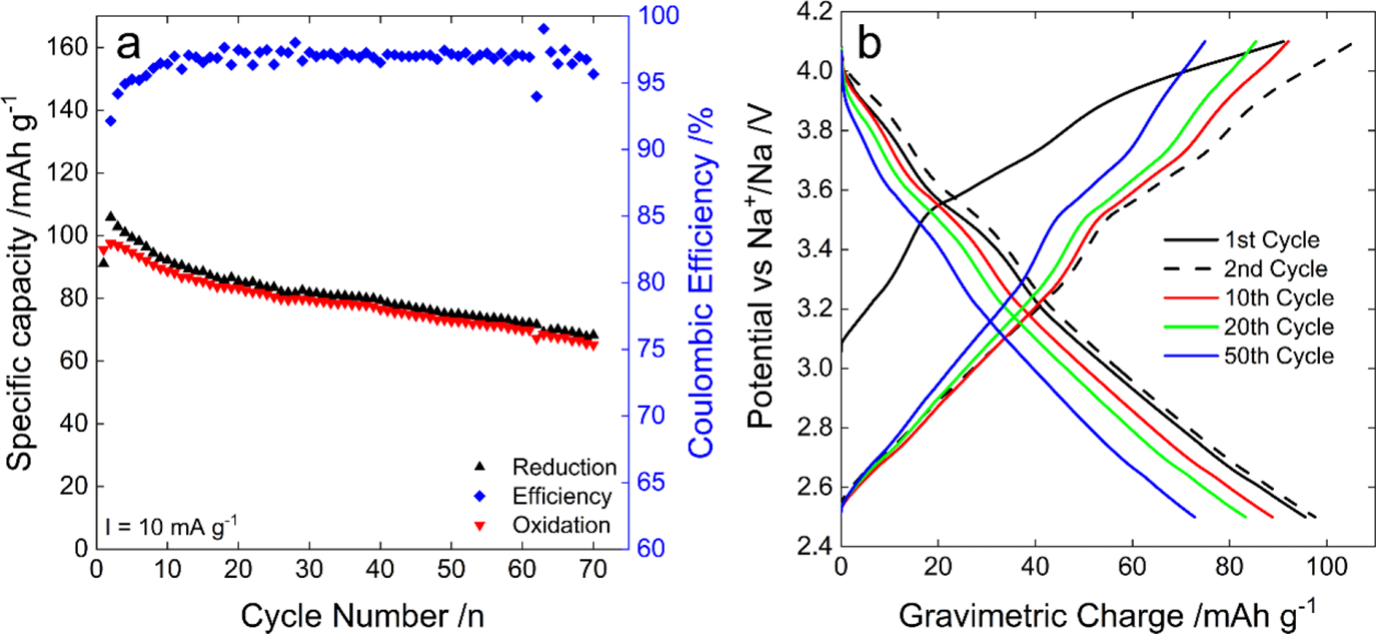
Electrochemical testing of NMNO_B in the 2.5–4.1 V region. (a) specific capacity evolution over 70 cycles, (b) potential vs charge profiles at different cycle numbers

Based on the above-reported results, to improve the stability of NMNO_B, 2.5 and 4.1 V were chosen as cutoff potential values for the reduction and oxidation process, respectively. The results obtained in this narrower potential window are displayed in Figure 6. As expected, the specific capacity obtained in the first reduction process is lower (95 mAh g^–1^, as shown in Figure 6a) than that achievable with a wider potential window with no significant differences in the potential–charge profiles (comparing the oxidation/reduction curves shown in Figures 4d and 6b). Conversely, by narrowing the potential window, the stability of the material was slightly improved. After 70 cycles, the electrode still delivers a specific capacity of 65 mAh g^–1^, corresponding to a capacity retention of 68% (against 49% in the wider potential range) and capacity loss of only 0.43 mAh g^–1^ per cycle. In this case, the mean operating potentials are 3.53 V for the oxidation process and 2.75 V for the reduction one. However, the Coulombic efficiency of the process (only 97% average value, calculated by excluding the first cycle) represents a serious concern.

#### Diffusion Coefficient Measurement

**Figure 7:**
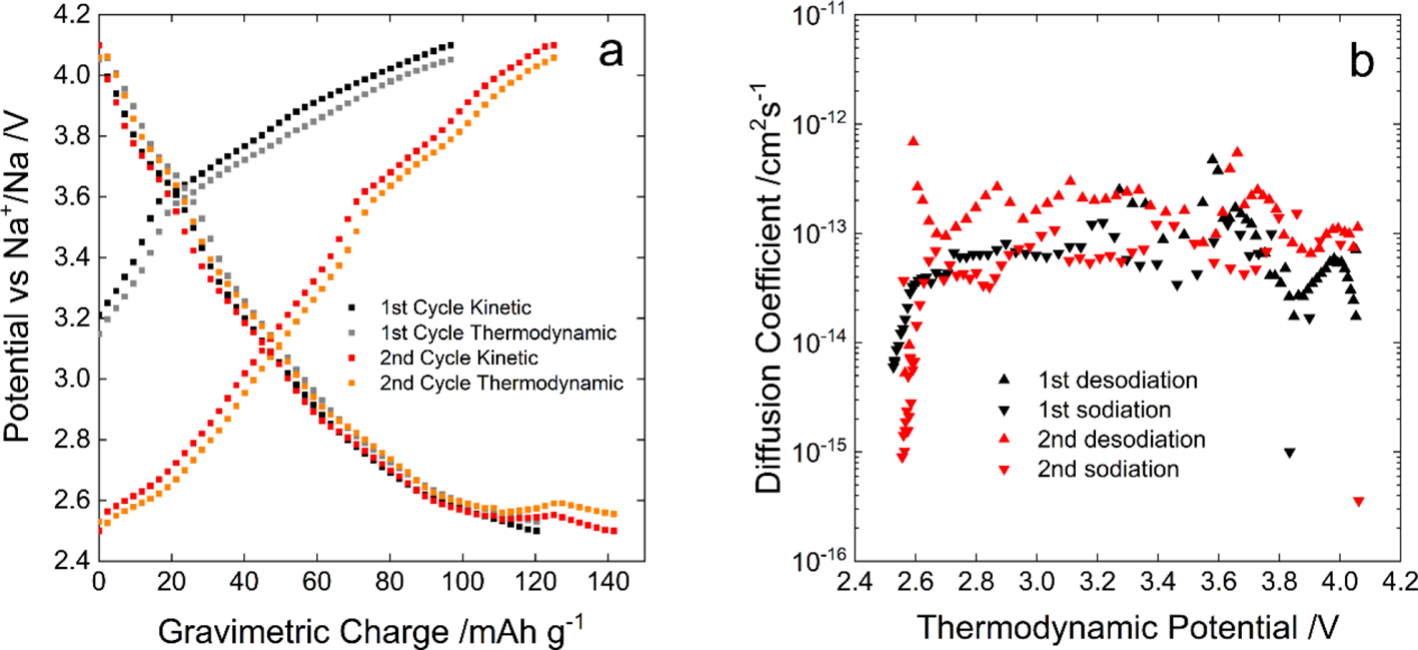
GITT analysis of NMNO_B (a) and the calculated diffusion coefficients (b).

The electrochemical performance of NMNO_B in the 2.5–4.1 V potential window was also evaluated by means of GITT (Figure 7). The Na^+^ diffusion coefficients calculated from the profiles shown in Figure 7a (in the order of 10^–14^ to 10^–15^ cm^2^ s^–1^, apart from the first values of the reduction process, as shown in Figure 7b) are comparable with those of many other layered oxides^68−70^ that typically exhibit high sodium diffusion due to their structure particularly favorable to Na-ion intercalation. From GITT analysis, it further came out that, at a current density of 10 mA g^–1^, the material exhibits a very low overpotential (40 mV), which demonstrates the good kinetic properties of the material. The values of the diffusion coefficients of sodium in the quasi-pristine NMNO_A (Figure S5) and NMNO_B (i.e., the value obtained after extraction of a negligible amount of sodium) were found to be 8.0 × 10^–14^ and 6.1 × 10^–13^ cm^2^ s^–1^. This growing trend is in line with that of the values of the displacement factors refined from Neutron diffraction data (3.9 and 4.9 Å^2^ form NMNO_A and NMNO_B respectively).

As for the parameters used for the determination of Na-ion diffusion coefficient, the tap density results to be 1.9 g cm^–3^, a value comparable both with those present in the literature for high-tap density material and that of the commercial standards for lithium (tap density of commercial lithium nickel manganese oxide ≥2.1 g cm^–3^).^71−73^

#### DFT Results

**Figure 8:**
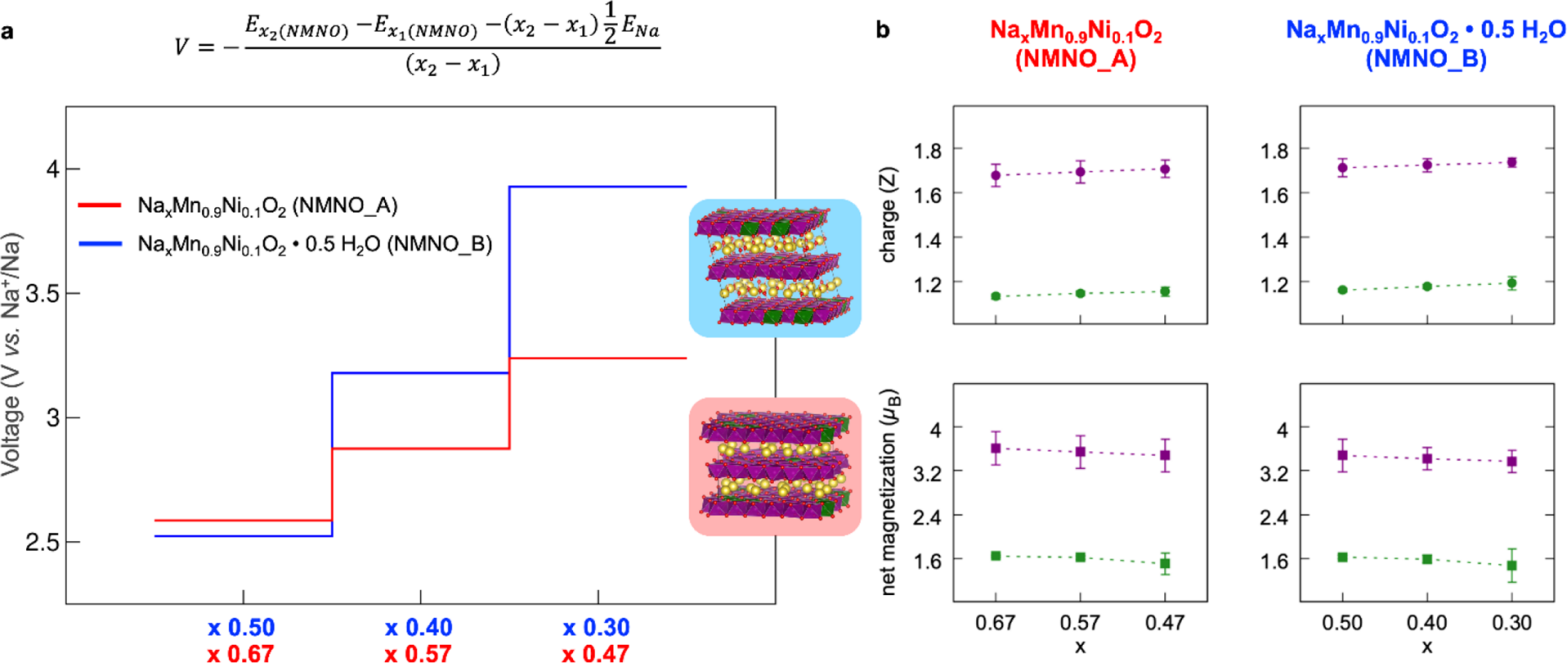
(a) Capacity-voltage profile computed at the PBE+U(-D3BJ) level of theory for NMNO_A (red) and NMNO_B (blue) as the sodium intercalation potential, defined in the displayed equation, for each *x* composition; (b) Bader charges (top) and net magnetizations (bottom)obtained for Mn and Ni sublattices in NMNO_A and NMNO_B at different Na contents.

The pristine and hydrated materials (namely, NMNO_A and NMNO_B) have been modeled as Na_0.67_Mn_0.9_Ni_0.1_O_2_ and Na_0.5_Mn_0.9_Ni_0.1_O_2_ · 0.5 H_2_O stoichiometries, which comply with the nominal compositions derived from the synthesis and further confirmed by elemental analysis. The desodiation process occurring upon cathode charge has been simulated by varying the Na content, *x*, within the parent structures. In particular, *x* = 0.67, 0.57, 0.47 compositions have been investigated for the P2 phase and *x* = 0.50, 0.40, and 0.30 for the birnessite one, both corresponding to the subsequent extraction of 6 Na atoms within the employed supercells. As listed in Figure S1b in SI, the theoretical data confirm the expansion of the *c*-parameter in the birnessite phase upon desodiation, while the P2 structure undergoes lattice compression along the *c*-direction. The theoretical capacity-voltage profile can be obtained by calculating the sodium intercalation potential as a function of the Na content (Figure 8), that is the energy required to extract a certain amount of Na from a given composition (see the equation plotted on top of Figure 8a). This represents a well-established method to predict the cathode behavior upon charge.^74−77^ The higher desodiation potential required for the hydrated phase compared to the pristine P2 is also validated by DFT results (see cyan and red lines in Figure 8a). Moreover, it is possible to couple each desodiation step to the oxidation process burdened by the TM sublattice. By calculation of charges and net magnetizations on Mn and Ni sublattices, the TM contribution to charge compensation has been dissected (see Figure 8b). Both phases feature very similar behavior in their electronic structures upon charge, with the mixed Mn^3+^/Mn^4+^ state undergoing further oxidation at the decreasing Na content (i.e., average magnetization decreases from 3.61 μ_B_ to 3.48 μ_B_ in NMNO_A and from 3.48 μ_B_ to 3.37 μ_B_ in NMNO_B, which can be ascribed to d^4^ → d^3^ electronic configurations) and Ni^2+^ → Ni^3+^ (i.e., average magnetization decreases from 1.64 μ_B_ to 1.51 μ_B_ in NMNO_A and from 1.63 μ_B_ to 1.47 μ_B_ in NMNO_B, which can be ascribed to d^8^ → d^7^ electronic configurations). The decreasing magnetization trend is coupled to a smooth increase in Bader charges, the values being far from the ionic limit owing to the highly covalent character of TM–O bonds. As a matter of fact, the unchanged Mn and Ni oxidation states within the water-free and water-containing structures would suggest that water uptake is not leading to any inferred material oxidation, and thus, the lower Na occupation degree in the birnessite structure may solely rely on the less available crystalline sites that are partially occupied by water molecules.

**Figure 9:**
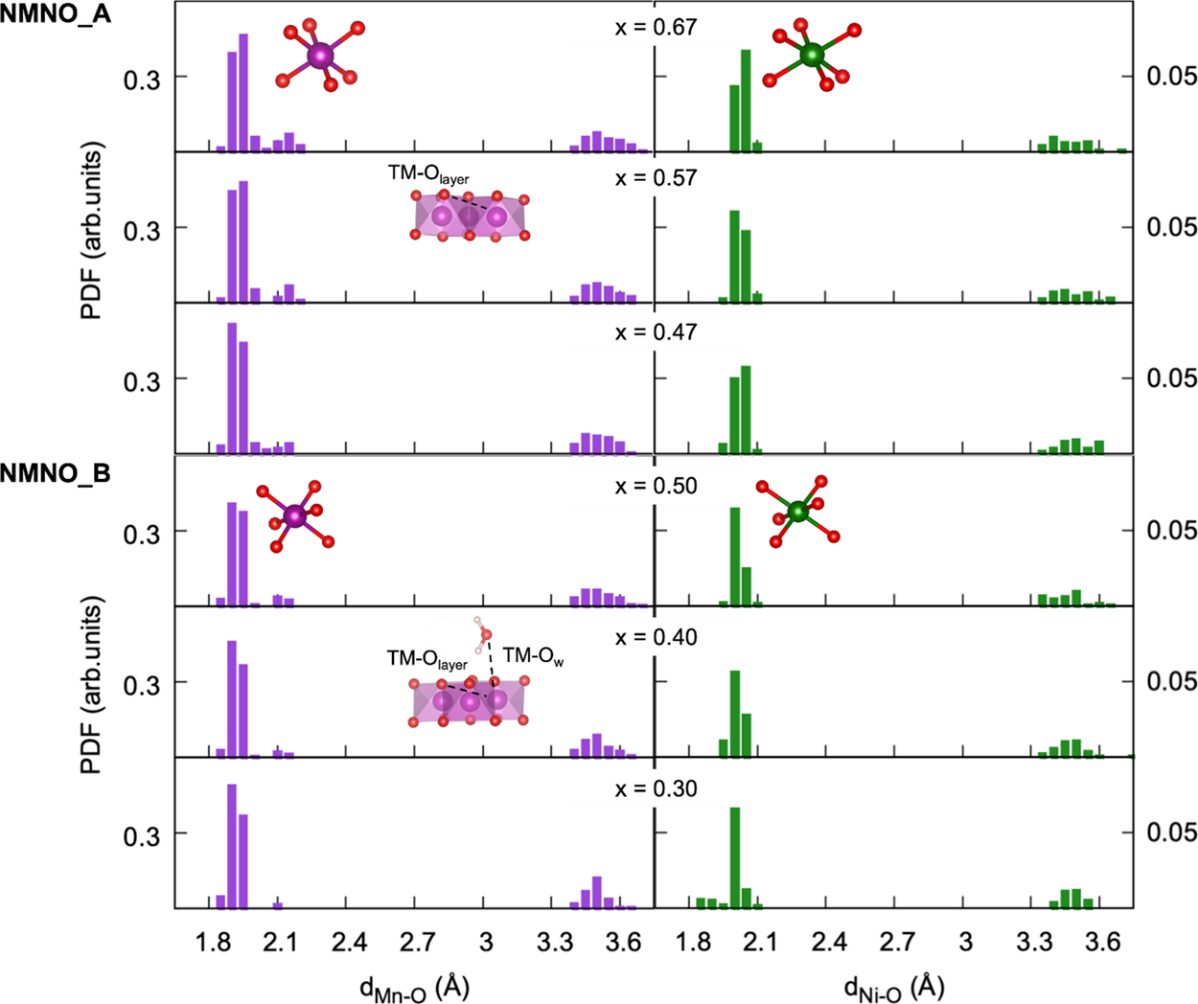
Pair distribution functions of Mn–O (purple) and Ni–O (green) distances in NMNO_A (top) and NMNO_B (bottom) at different Na contents (*x*). Structural details are displayed in the graphs to highlight the most significant distance ranges: the TM–O bond length within the TMO_6_ octahedra and the TM–O_w_/TM–O_layer_ inter/intralayer distances.

Structural analysis upon desodiation is investigated in terms of pair distribution functions (PDFs) of TM–O and Na/H–O distances, so as to dissect each inter- and intralayer coordination. This purely computational analysis aims at determining to what extent each atom pair (i.e., TM–O, Na–O, and H–O) specifically contributes to structural rearrangements affecting the materials upon desodiation. By looking at TM–O PDFs in Figure 9, a more ordered structuring within the TMO_6_ octahedra can be detected in the NMNO_B material, where the narrower short-range peaks at ∼2 Å indicate less spread bond lengths. For both phases, the left-shifting trend of the short-range distances at decreasing Na content suggests a general compression of the TMO_6_ octahedra, following the TM oxidation occurring upon desodiation. This result is in line with the decreasing/increasing magnetization/charge trends already revealed from the electronic structure analysis (Figure 9a). The long-range peaks at ∼3.5 Å are indicative of the TM–O interactions within the second coordination shell. It is worth mentioning that in the hydrated phase, the TM–O_w_ distances fall in the same range, suggesting that similar metal–oxygen interactions are established either as intra- and interlayer (TM–O_layer_ and TM–O_w_ labeled in Figure 9).

**Figure 10:**
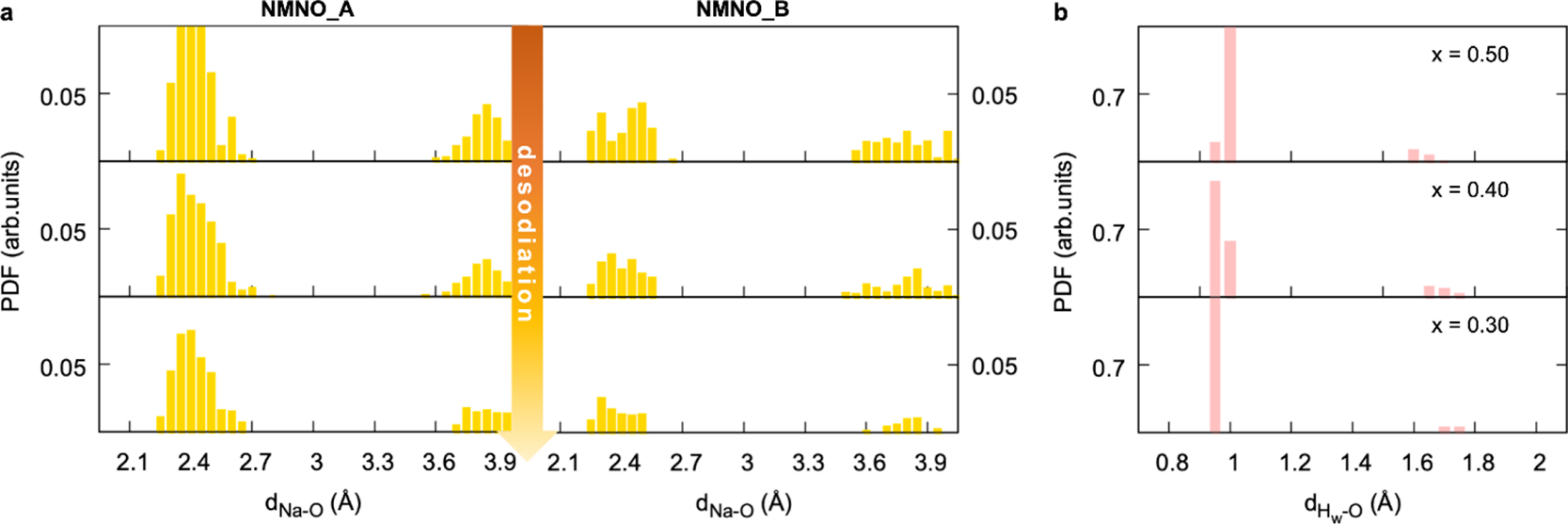
Pair distribution functions of (a) Na–O distances (yellow) in NMNO_A and NMNO_B, and (b) H–O distances (pink) in NMNO_B plotted at different Na contents.

Further insights into intra- and interlayer structuring can be gained from the Na–O PDFs reported in Figure 10. In NMNO_B, Na coordination is saturated by oxygen atoms not only from the TMO_2_ sheet but also from the water oxygens lying in the same layer. At a lower Na content, such Na–O interactions become tighter, as suggested by the left-shift of the short-range peaks detectable in Figure 10a. Finally, the H–O PDFs (Figure 10b) indicate that desodiation leads to water bonds (H_w_–O_w_, at ∼1 Å) shortening and water-layer (H_w_–O_layer_, at ∼1.7 Å) enlargement, which can be ascribed to the increased electrostatic repulsion also leading to the expansion of interlayer spacing (higher *c*-parameter). All-in-all, it is possible to conclude that the intercalated water in the birnessite phase can establish favorable interactions with the TMO_2_-layer, even at decreasing sodium content, thus explaining the underlying structural stability that is still retained upon desodiation. The water uptake acts as a balance toward the unsaturated Na–O coordination induced by the expanded c-parameter and determines the structural stability of the birnessite phase that was previously evidenced by the Karl Fischer experiment.

## Conclusions

This work demonstrates that, by simply exposing to air (i.e., moisture) a cathodic P2-type layered oxide Na_*x*_Mn_0.9_Ni_0.1_O_2_, a new crystalline phase with Na-rich birnessite structure can be obtained, whose specific microscopic feature is the intercalation of orientationally ordered water molecules between the (Mn/Ni)O_6_ layers. This orientational order leads to doubling of the unit cell along the *c*-axis, and we propose a model for the crystal structure with space group *C*2/*c*. Experimental evidence of air-exposing stability for such Na_*x*_Mn_0.9_Ni_0.1_O_2_ cathode is disclosed from first-principles: electrode contact with moist air leads to the formation of a birnessite phase, where water molecules can establish favorable interactions with the TMO_2_-layer (i.e., the interlayer O_w_–TM distances fall in the same range of intralayer O_layer_–TM ones that are also present in the water-free material). The presence of intercalated water can balance the unsaturated Na–O coordination induced by the expanded *c*-parameter. Despite storing a lower amount of sodium due to less available crystalline sites that are partially occupied by water molecules, the hydrated material is still capable of good electrochemical performance. In fact, such a new phase is able to deliver a specific capacity of ∼100 mAh g^–1^ with a capacity loss of only 0.43 mAh g^–1^ per cycle in the 2.5–4.1 V vs Na^+^/Na window, in line with the typical behavior of many Mn-rich layered oxides cathodes.^22,27,78−80^ As unveiled by DFT results, water-free and water-containing materials share the same charge compensation mechanism, with Mn^3+^/Mn^4+^ and Ni^2+^/Ni^3+^ being the redox active couples upon desodiation. These electronic features, together with the structural changes discussed within experimental investigations and theoretical simulations, can explain the similar electrochemical behavior observed upon charge. While overall the cycling performances of the hydrated phase are slightly worse than that of the pristine one, the higher diffusion coefficient makes it of potential interest for some mid-to-high power applications.

## Supporting Information Available

Details about the model used for DFT calculations; refined parameters from diffraction analyses; table of the results of elemental analyses; TGA figure of NMNO_A; SEM analyses on electrodes; and results of GITT and diffusion coefficient of NMNO_A (PDF)
