# Supplementary material for: Structural Evolution of Air-Exposed Layered Oxide Cathodes for Sodium-Ion Batteries: An Example of Ni-doped NaxMnO2
Source: Chem Mater. 2023 Oct 11;35(20):8440–54. doi: 10.1021/acs.chemmater.3c01196 (PMC10601480; doi:10.1021/acs.chemmater.3c01196)
Supplement: Supplementary file 1 — cm3c01196_si_001.pdf [file cm3c01196_si_001.pdf]

Supporting Information

for

On the structural evolution of air-exposed layered oxide cathodes for sodium-ion batteries: the example of the Ni-doped Na_xMnO_2

AUTHOR NAMES: Gabriele Brugnetti[a], Claudia Triolo[b,d,e], Arianna Massaro[c,d], Irene Ostroman[a], Nicolò Pianta[a], Chiara Ferrara[a,d,e], Denis Sheptyakov[f], Ana Belén Muñoz-García[d,g], Michele Pavone[c,d], Saveria Santangelo*[b,d,e] and Riccardo Ruffo[a,d,e]*

AUTHOR ADDRESS: a. Dipartimento di Scienza dei Materiali, Università di Milano Bicocca, Milano 20125, Italy.

b. Dipartimento di Ingegneria Civile, dell'Energia, dell'Ambiente e dei Materiali (DICEAM), Università "Mediterranea", Via Zehender, Loc. Feo di Vito, 89122 Reggio Calabria, Italy.

c. Dipartimento di Scienze Chimiche, Università di Napoli Federico II, Napoli 80126, Italy.

d. National Reference Center for Electrochemical Energy Storage (GISEL), Via G. Giusti 9, Firenze 50121, Italy

e. Consorzio Interuniversitario per la Scienza e Tecnologia dei Materiali (INSTM), Via G. Giusti 9, Firenze 50121, Italy

f. Laboratory for Neutron Scattering and Imaging, Paul Scherrer Institut, 5232 Villigen PSI, Switzerland

g. Dipartimento di Fisica "E. Pancini", Università di Napoli Federico II, Napoli 80126, Italy.

Structural models for DFT calculations

The 220 atoms-containing 5x3x1 supercell of $Na_{0.67}Mn_{0.9}Ni_{0.1}O_2$ (P2, NMNO_A) and the 300 atoms-containing 3x5x1 supercell of $Na_{0.5}Mn_{0.9}Ni_{0.1}O_2$ • 0.5 H_2O (birnessite, NMNO_B) are showed in Figure S1a. The lattice parameters within the $P6_3/mmc$ and the C2/c space group, respectively, have been computed at the PBE+U(-D3BJ) level of theory. The values listed in the table of Figure S1b show a ~2% and ~0.5 % deviation from experimental data for NMNO_A and NMNO_B, respectively, on each lattice constant.

a)

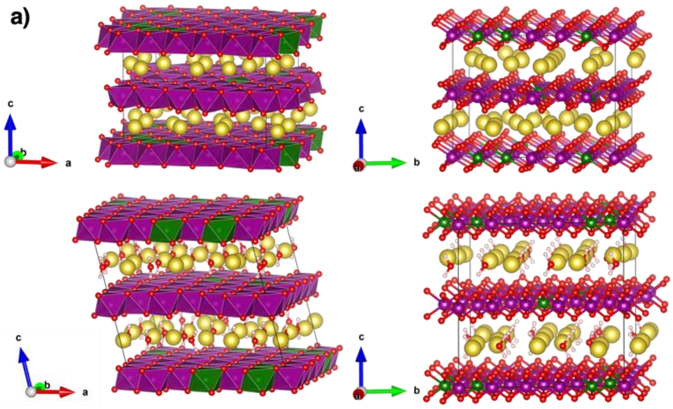

b)

NMNO_A	a (Å)	b (Å)	c (Å)
x Na 0.67	14.652	15.258	11.422
x Na 0.57	14.652	15.258	11.422
x Na 0.47	14.610	15.214	11.389

NMNO_B	a (Å)	b (Å)	c (Å)	β (°)
x Na 0.50	15.117	14.522	14.528	103.343
x Na 0.40	15.130	14.533	14.539	103.343
x Na 0.30	15.138	14.541	14.547	103.343

Figure S1 – Structural models employed for DFT calculations: a) side views of the considered supercells and b) lattice parameters computed at PBE+U(-D3BJ) level of theory for (top) pristine, $Na_{0.67}Mn_{0.9}Ni_{0.1}O_2$ (NMNO_A), and (bottom) hydrated materials, $Na_{0.5}Mn_{0.9}Ni_{0.1}O_2$ • 0.5 H_2O (NMNO_B). Atoms are represented as spheres. Colour code: Na (yellow), Mn (purple), Ni (green), O (red), H (pink). TMO_6 octahedra are highlighted in the structure representations on the left side.

Refined parameters from diffraction data analysis

Table S1 – Structural parameters and agreement factors obtained for refinements of powder diffraction neutron data for NMNO_A and NMNO_B samples. Error in the total occupancy value for each species can be estimated in 5%.

**NMNO_A sample - P2 structure S.G. $P6_3/mmc$**				
**a / Å**	**b / Å**	**c / Å**		
2.87161(25)	2.87161(25)	11.18154(106)		
	**x**	**y**	**z**	**occ**
**Na1**	0.3333	0.6667	0.75	0.11
**Na2**	0	0	0.25	0.29
**Mn/Ni**	0	0	0	0.87 / 0.13
**O**	0.3333	0.6667	0.5898(2)	1
Rwp: 7.20; Chi2: 1.43				

**NMNO_B – Birnessite structure S.G. C 2/c**				
**a / Å**	**b / Å**	**c / Å**	**β / deg**	
5.01208 (84)	2.89406 (41)	14.45890 (187)	103.23717 (1025)	
	**x**	**y**	**z**	**occ**
**Na**	0.5	0.386 (5)	0.25	0.46
**Mn/Ni**	0	0	0	0.89/0.11
**O**	0.121 (2)	0.504 (2)	0.4296 (2)	1
**Ow**	0	0.307 (3)	0.25	0.44
Rwp: 1.33; Chi2: 2.51				

Table S2 – Structural parameters and agreement factors obtained for refinements of powder diffraction ex situ data for the ex-situ NMNO_B samples analysed through traditional Rietveld refinements and Faults software, as described in the Experimental method.

	**a / Å**	**b / Å**	**c / Å**	**β / deg**	**Rwp**	**Chi2**
**NMNO_B_pe Riet. Ref.**	5.0204(34)	2.8974(17)	7.2241(29)	103.1803(25)	0.771	5.30
**NMNO_B_es20 Riet. Ref.**	5.0149(29)	2.8950(16)	7.26257(23)	103.2314(76)	0.821	6.12
**NMNO_B-es60 Riet. Ref.**	4.9996(32)	2.8842(18)	7.3189(31)	103.2888(58)	0.907	7.31
**NMNO_B-es60 Faults Ref.**	4.9824(96)	2.875 (72)	7.301 (14)	103.253 (45)	0.911	5.89

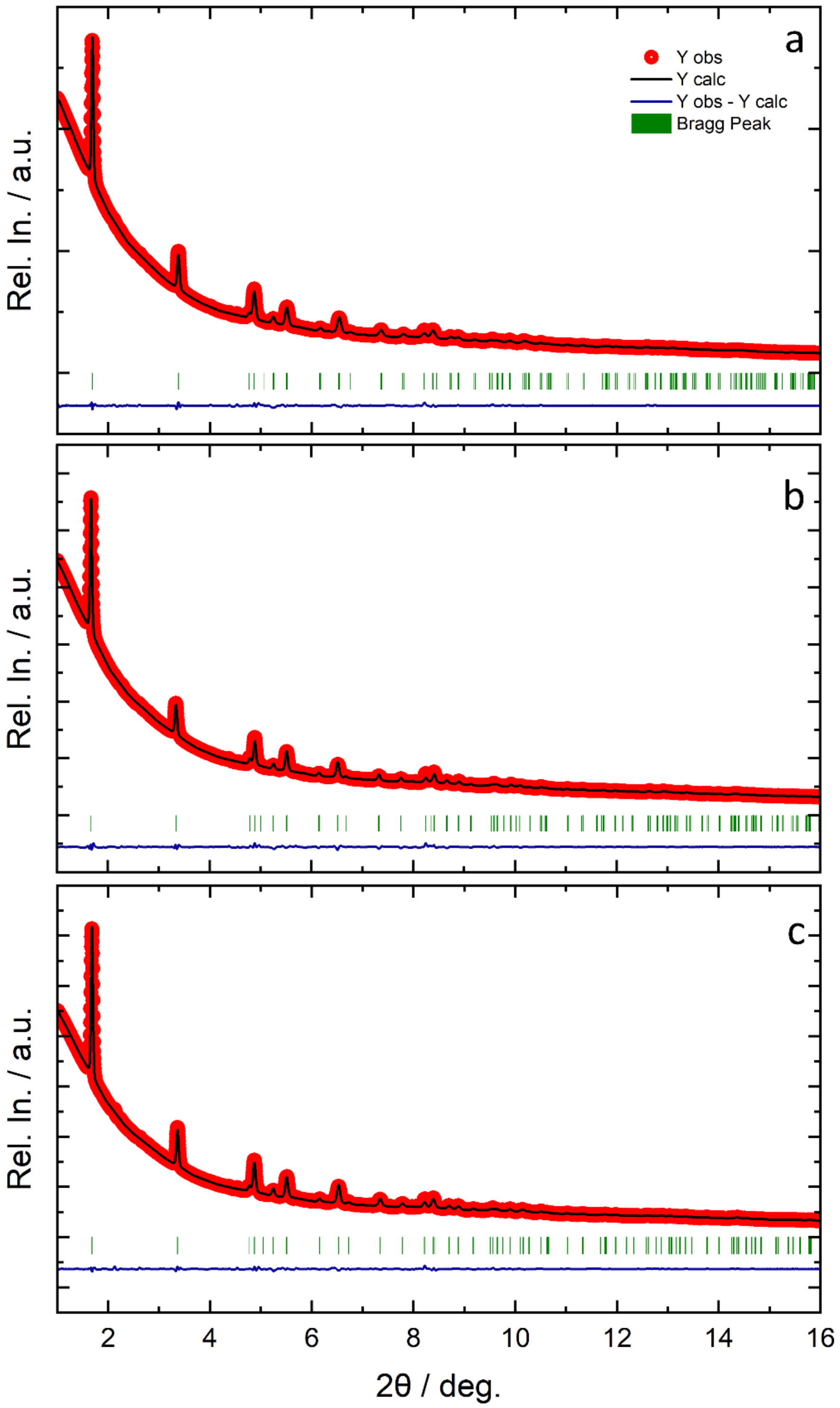

Figure S2 – Rietveld refinements for ex situ XRD data obtained for selected NMNO_B samples: NMNO_B_pe (a), NMNO_B_es20 (b), NMNO_B_es60 (c).

Elemental analysis results

Table S3 – Stochiometric coefficients of sodium, manganese and nickel measured through ICP and mass percentages of hydrogen and carbon measured through CHNS for NMNO_A and NMNO_B

**Stochiometric Coefficients**	**Na**	**Mn**	**Ni**	**Mass Percentage**	**H**	**C**
**NMNO_A**	**0.67**	**0.9**	**0.1**	**NMNO_A**	**0.144**	**1.25**
**NMNO_B**	**0.52**	**0.9**	**0.1**	**NMNO_B**	**1.38**	**1.26**

Thermal analysis on NMNO_A

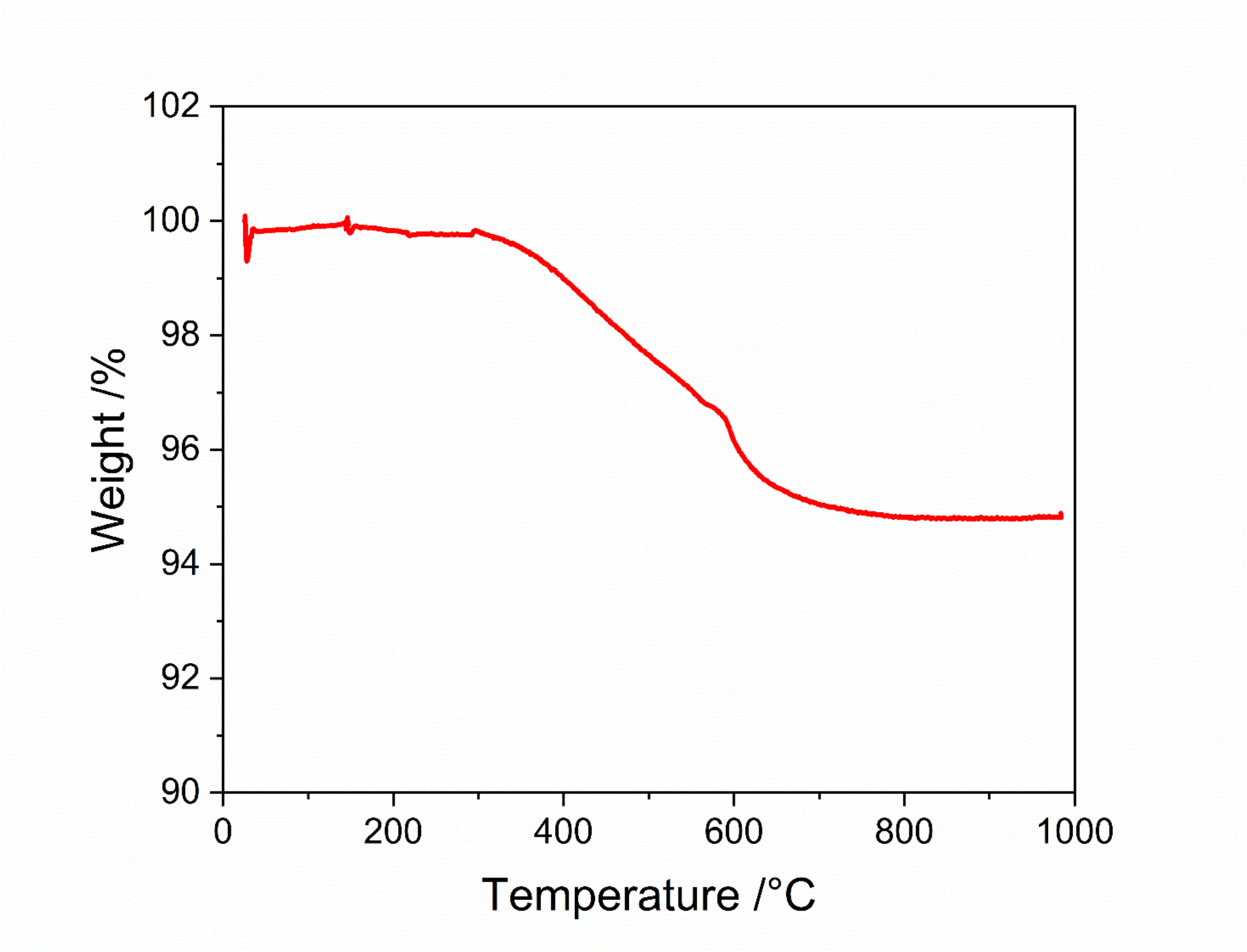

Figure S3 – Weight loss vs temperature plot extracted from TGA analysis of pristine NMNO_A powders.

SEM analysis on electrodes

All electrode components are visible in the SEM images, namely conductive carbon and binder, both with nearly spherical shape and size around 50 nm, and active material particles featured by regular edges, as in the pristine material, which proves their unaltered crystallinity. No evidence of delamination is detected in the samples.

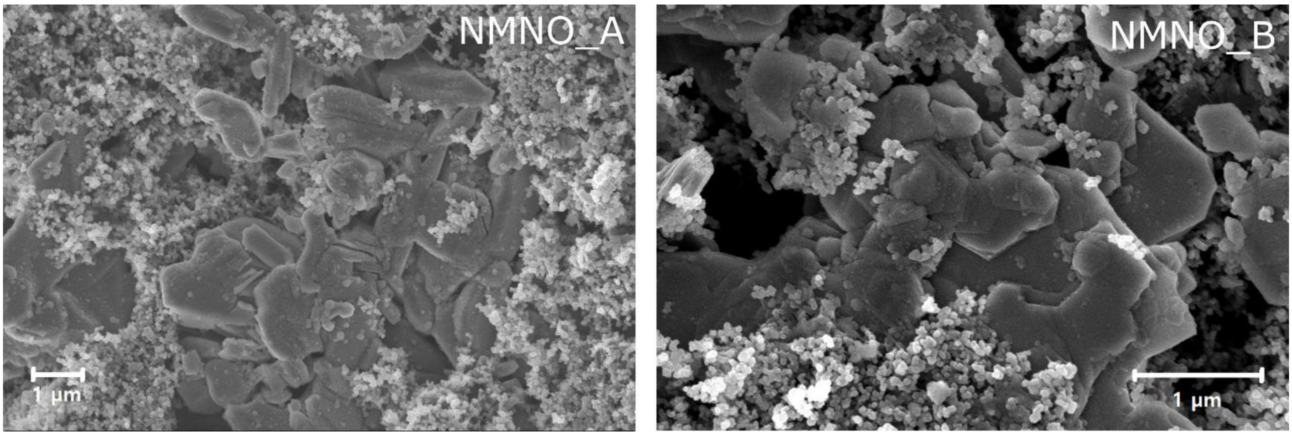

Figure S4 – SEM images of the disassembled electrodes

Diffusion coefficient of NMNO_A

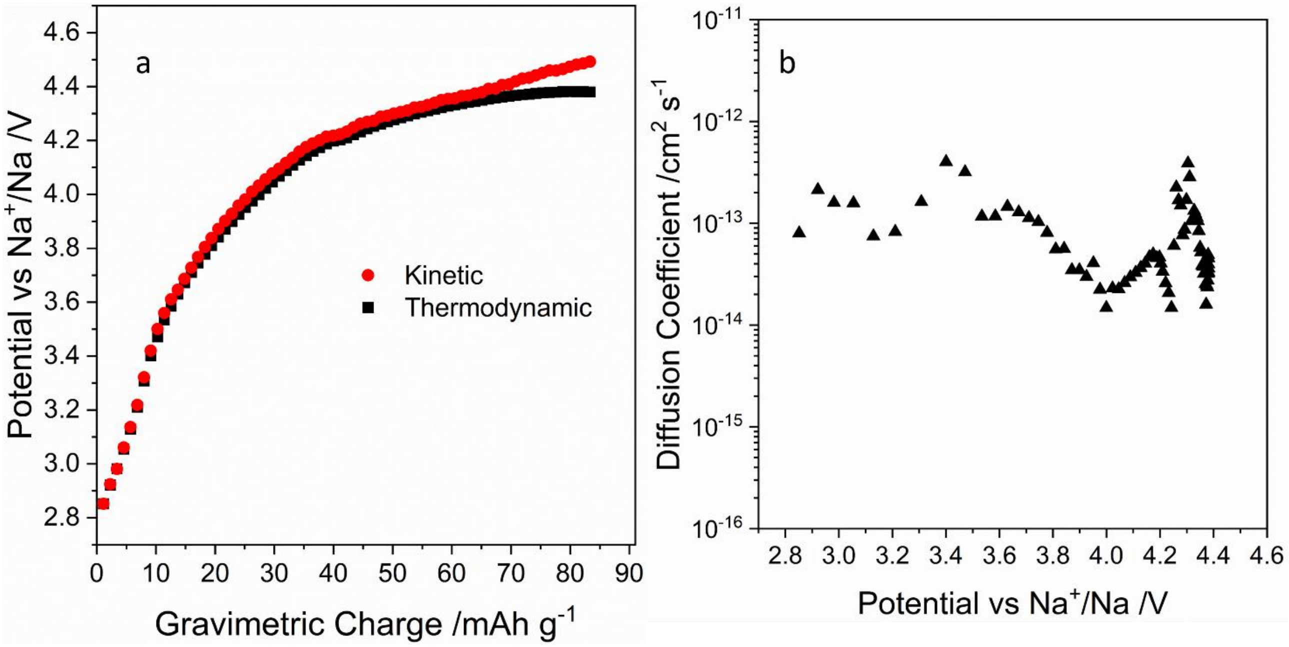

Figure S5 – GITT results for the first oxidation of NMNO_A. Kinetic and Thermodynamic potentials vs Gravimetric Charge (a) and value of calculated diffusion coefficient (b).
